# Real-life speech production and perception have a shared premotor-cortical substrate

**DOI:** 10.1038/s41598-018-26801-x

**Published:** 2018-06-11

**Authors:** Olga Glanz (Iljina), Johanna Derix, Rajbir Kaur, Andreas Schulze-Bonhage, Peter Auer, Ad Aertsen, Tonio Ball

**Affiliations:** 1 GRK 1624 ‘Frequency Effects in Language’, University of Freiburg, Freiburg, Germany; 2 Department of German Linguistics, University of Freiburg, Freiburg, Germany; 3 Hermann Paul School of Linguistics, University of Freiburg, Freiburg, Germany; 4 Translational Neurotechnology Lab, Department of Neurosurgery, Medical Center - University of Freiburg, Faculty of Medicine, University of Freiburg, Freiburg, Germany; 5 BrainLinks-BrainTools, University of Freiburg, Freiburg, Germany; 6 Neurobiology and Biophysics, Faculty of Biology, University of Freiburg, Freiburg, Germany; 7 Faculty of Medicine, University of Cologne, Cologne, Germany; 8 Epilepsy Center, Department of Neurosurgery, Medical Center - University of Freiburg, Faculty of Medicine, University of Freiburg, Freiburg, Germany; 9 Bernstein Center Freiburg, University of Freiburg, Freiburg, Germany

## Abstract

Motor-cognitive accounts assume that the articulatory cortex is involved in language comprehension, but previous studies may have observed such an involvement as an artefact of experimental procedures. Here, we employed electrocorticography (ECoG) during natural, non-experimental behavior combined with electrocortical stimulation mapping to study the neural basis of real-life human verbal communication. We took advantage of ECoG’s ability to capture high-gamma activity (70–350 Hz) as a spatially and temporally precise index of cortical activation during unconstrained, naturalistic speech production and perception conditions. Our findings show that an electrostimulation-defined mouth motor region located in the superior ventral premotor cortex is consistently activated during both conditions. This region became active early relative to the onset of speech production and was recruited during speech perception regardless of acoustic background noise. Our study thus pinpoints a shared ventral premotor substrate for real-life speech production and perception with its basic properties.

## Introduction

A basic and intuitive assumption of communication theories is that the sender and the recipient of a message rely on shared cognitive resources to be able to understand each other^1^. In agreement with this idea, psychophysiological theories of language assume a physiological link between natural speech production and perception. The articulatory system has been proposed as a candidate to support both phenomena and to either enable^2,3^ or at least facilitate^4,5^ comprehension by making use of articulatory knowledge during speech perception. Several theories are situated within this general motor-cognitive framework, including the motor theory of speech perception (MTSP)^2,3,6^, analysis-by-synthesis^7^, direct realism^8,9^, and embodied language cognition^5,10,11^. All of these are compatible with the notion of a spatial overlap between speech production and perception in the articulatory cortex. The revised version of the MTSP^3^ further assumes that articulatory intentions are the shared motor object of speech production and perception. From this assumption, the hypothesis can be derived that activity in the putative speech-production-perception-overlap region in the motor cortex will take place very early in the time course of speech production.

The theoretically predicted motor-cortical area activated during speech perception has received much attention in neuroscientific research over the last 15 years, leading to controversial empirical results. While some studies did not observe such an area at all^12^, others found it only in subjects with particularly good short-term memory^13^, or with especially high proficiency in a second language^14^, still others reported reproducible motor-cortical activation during auditory^15,16^, visual^17,18^, and audiovisual^19–21^ tasks. In spite of several converging lines of evidence supporting the involvement of the motor cortex in perceptual processes, concerns have been raised as to whether positive findings might be an artefact of unnatural, experimental conditions of investigation, rather than being representative of natural language processing^22,23^. Indeed, there is evidence that activation of the motor cortex during speech perception can be modulated by the experimental paradigm, such as by an increased level of attention in cognitively demanding tasks^24^, or by the subjects’ expectations about the upcoming stimuli^25,26^.

Previous studies addressing the putative overlap between speech perception and production in the articulation-relevant cortex had generated results that divided researchers into two camps: Those who postulate, in line with the MTSP^2,3,6^, that activity in the articulatory cortex during speech perception must be necessary for comprehension, and those who consider that such activity is a rather exotic phenomenon that hardly ever occurs at all. The latter camp raised concerns that observations of activity in the motor cortex during perception can merely be an artifact of experimental procedures, such as due to unnaturally high attentional demands during experiments^22^. Researchers who sympathize with the former idea, on the contrary, have suggested that the absence of motor-cortical activity during speech perception may be due to unnaturally high levels of background noise during fMRI scanning^27^. A hitherto unresolved question resulting from this debate is whether or not activity in the articulatory cortex is a robust feature of natural speech perception that occurs regardless of experimental manipulations. This question can only be studied by taking the research from experiments to out-of-the lab conditions of human communication.

Examination of cortical activity in real-life contexts of communication, however, presents a formidable methodological challenge. Freely behaving humans can hardly be studied within the confined space of functional magnetic resonance imaging (fMRI), positron emission tomography (PET) or magnetoencephalography (MEG) scanners. Although non-invasive electroencephalography (EEG) allows for more unrestricted behavior, investigation of non-experimental communication with this method can be difficult due to its vulnerability to movement-related artefacts^28^ that are invariantly associated with natural communication. In contrast, intracranially-recorded electrocorticography (ECoG) combines high spatial and temporal resolution^29^, and enables assignment of neuronal responses to anatomical areas based on MRI data^30^. Additionally, it is more resistant against artefacts, and has a higher signal-to-noise ratio than non-invasive EEG^31^. These properties make ECoG a unique tool to study motor cognition and social interaction in conditions of naturalistic, non-experimental behavior^28,30,32,33^. In the present study, we used ECoG recordings to clarify whether cortical motor areas involved in speech production are activated in speech perception during real-world communication.

We recorded ECoG data directly from the cortical surface of epilepsy patients while they were engaged in spontaneous, everyday conversations with visitors or medical staff, and investigated high-gamma activity accompanying speech production and perception in several frequency bands (70–100, 100–200, and 200–350 Hz). Previous ECoG research has shown that high-gamma activity is a robust marker of event-related cortical activation^32,34,35^ that is thought to reflect cortical spiking processes^34,36^. High-gamma activity occurs in cortical areas during speech production^37,38^ and perception^38^ and can be observed in relation to language in instructed experiments as well as during non-experimental, real-world communication^28,30,32,33^. Since the classical functional definition of the motor cortex is based on its electrical excitability in direct electrocortical stimulation mapping (ESM)^39^, we used ESM to determine whether the anatomical motor cortex with overlapping activity between speech production and perception we identified with ECoG had mouth motor properties.

Once we had established that an overlap between neuronal activation during speech production and perception in the mouth motor cortex in our data was highly reproducible, we undertook additional analyses to describe this overlap area in more detail. Different potential roles for the involvement of the motor cortex in receptive speech have been proposed. Amongst others, a hypothesis exists that enhanced levels of motor-cortical activity during speech perception may be a signature of predictive coding used to facilitate extraction of communication-relevant information from a noisy acoustic signal^40^. Experimental reports are divided on whether the motor cortex is engaged only when subjects attend to distorted speech^41^ or also in conditions of clear-speech perception^24^. To address the dependency of cortical activation on acoustic background noise, we compared the level of cortical activation during listening to clear speech with listening to speech in the presence of strong acoustic background noises and with listening to non-speech noises.

The present study addressed four main hypotheses: (i) There is an area in the anatomical motor cortex which is reproducibly activated during natural speech production and perception, (ii) this area has mouth motor properties, (iii) it shows preparatory neuronal activity related to speech production earlier than other cortical regions, and (iv) neuronal activity in this area during perception is modulated by the presence of strong acoustic background noises. Addressing these hypotheses may help validating controversial assumptions that emerged on the basis of previous experimental findings but that could not be tested until now because they refer to natural, non-experimental conditions of human communication.

## Results

### Overlapping activity between speech production and perception in the articulatory superior ventral premotor cortex (svPMC)

We combined ECoG and ESM in a within-subject design to account for both structural and functional properties of neuronal activity related to speech production and perception. On the surface of the anatomical motor cortex of all subjects (specifically, in the premotor cortex and on the adjacent central sulcus determined using hierarchical probabilistic assignment^30^, see Methods), we detected a focal region that showed reproducible ECoG responses in both speech production and perception. Interestingly, this region possessed mouth motor properties as defined by ESM. We will further refer to this anatomical-functional speech-production-perception-overlap region as ‘aSPPO’ (‘a’ for ‘articulatory’).

#### Assignment of electrodes to functional areas

**Figure 1:**
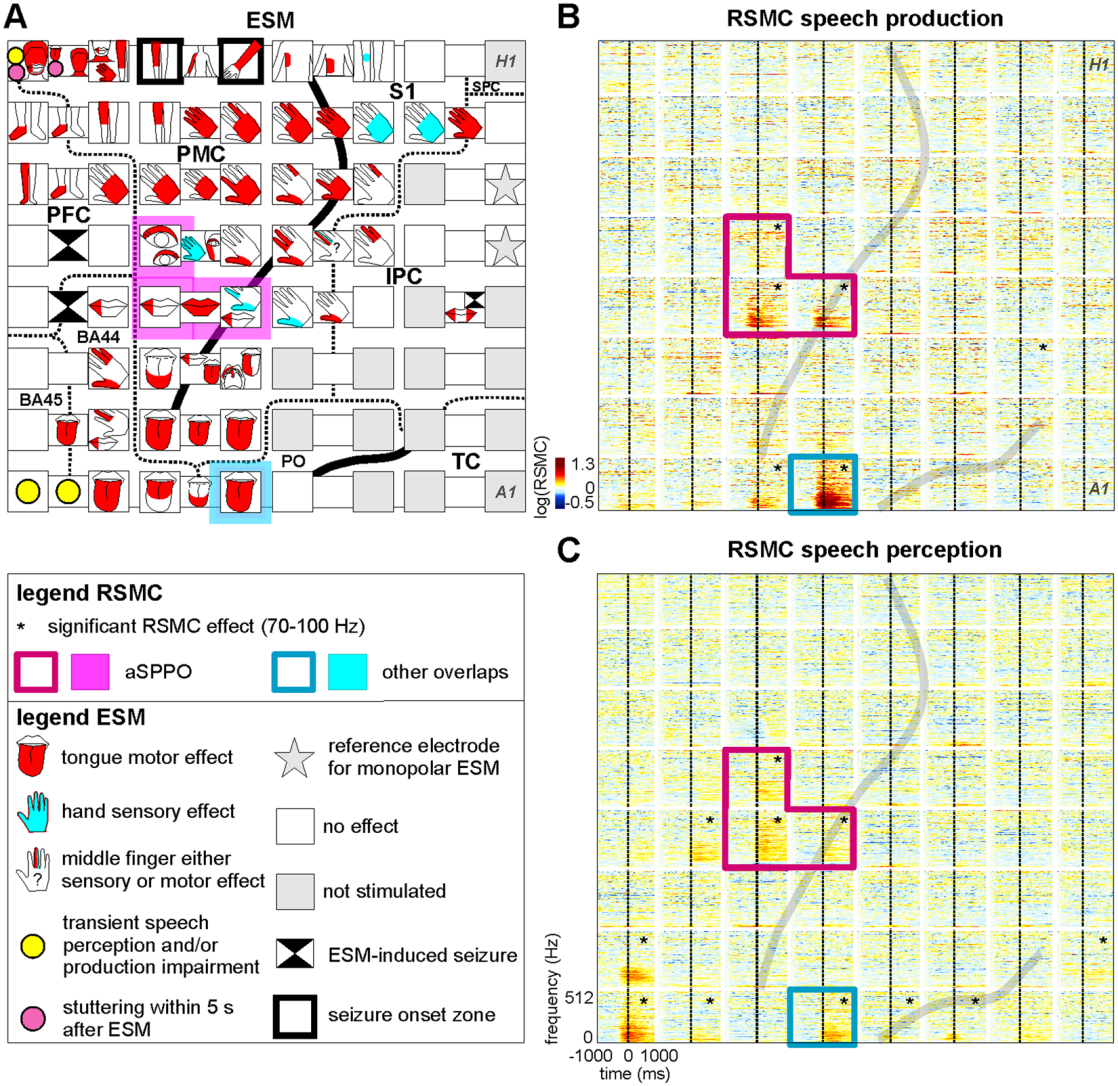
Typical electrocortical stimulation mapping (ESM) results and relative spectral magnitude changes (RSMC) during non-experimental, real-life speech (S2). (**A**) ESM results are visualized with reference to the individual anatomical areas of this subject. The borders of anatomical areas are marked by dotted lines. Black solid lines indicate positions of the central and lateral sulci. BA: Brodmann area, IPC: inferior parietal cortex, PFC: prefrontal cortex, PMC: premotor cortex, PO: parietal operculum, SPC: superior parietal cortex, S1: primary somatosensory cortex, TC: temporal cortex. The magenta overlay in A and the magenta boxes in B and C highlight the electrodes lying in both anatomically and functionally defined articulatory motor cortex (see Methods). This region, located in the anatomical motor cortex and showing mouth motor effects upon ESM, will be referred to as an articulatory speech-production-perception-overlap, ‘aSPPO’. The blue overlay in A and the blue boxes in B and C highlight the electrode with significant RSMC during both speech production and perception that lay outside the aSPPO region (‘other overlaps’ in the legend). *H1* to *A1*: electrode labels included for ease of spatial reference. (**B**) Time-frequency-resolved RSMC at the 8 × 8 electrode grid during speech production. Grey lines indicate the individual locations of the central (nearly vertical line in the middle part of the panel) and lateral (diagonal line in the lower right) sulci, log: natural logarithm. The black stars in the upper right corners of the individual electrodes indicate that the RSMC in the frequency range of 70–100 Hz were significant (two-tailed Wilcoxon sign test, false discovery rate (FDR)-corrected at q = 0.005) relative to a baseline period (see Methods). Other conventions as in A. (**C**) RSMC observed at the 8 × 8 electrode grid during real-life speech perception, all conventions as in B.

Inspection of stimulation protocols and the ESM-accompanying video materials allowed us to generate highly detailed functional maps (Fig. 1A and Suppl. Fig. 1). In line with the classical findings by Penfield and Boldrey^39^, we observed a reproducible somatotopic arrangement of sensory and motor responses in the pericentral cortex, with hand and arm representations localized ventrally to the representations of lower extremities and dorsally to electrodes in areas with mouth functions that included lip, tongue, and soft palate motor effects. We considered these areas as potentially relevant for articulation. More details on the outcomes of ESM are available in Suppl. ESM results.

#### Gamma-activity modulations during real-world speech production and perception

**Figure 2:**
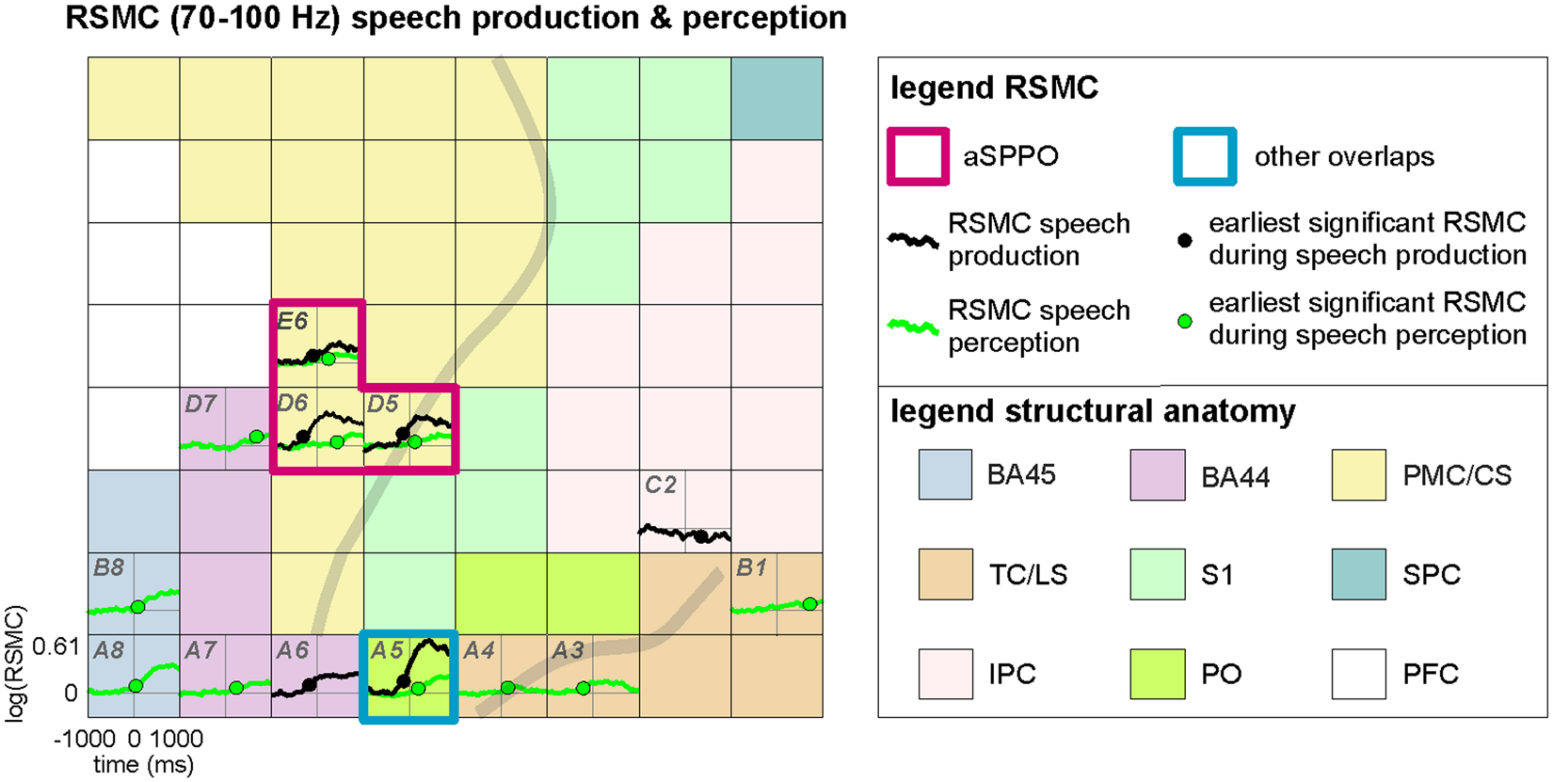
An example of typical frequency band-averaged RSMC during speech production and perception (S2, 70–100 Hz). Results of significant high-gamma RSMC (two-tailed Wilcoxon sign test, FDR-corrected at q = 0.005) for speech production (black solid curves) and perception (green solid curves) conditions are shown. Black and green dots on the curves indicate the earliest onsets of significant activation for the respective condition. Anatomical areas are color-coded, other conventions as in Fig. 1. Electrode labels (e.g., *H1*, *A1)* are included for all electrodes showing significant effects to facilitate spatial reference.

We wanted to test whether and in which anatomical areas high-frequency relative spectral magnitude changes (RSMC) underlying oral expressive and receptive speech overlapped during real-life communication. To this end, we identified the electrode sites at which significant high-gamma modulations (two-tailed Wilcoxon sign test, false discovery rate (FDR)-corrected^42^ at q = 0.005) were present in both conditions. Figure 1B,C illustrate the typical frequency-resolved RSMC during speech production and perception on the example of S2. Spatial overlaps of significant RSMC were inspected in several high-gamma frequency bands (70–100 Hz, 100–200 Hz, and 200–350 Hz, see Methods). Figure 2 provides a visualization of typical band-averaged RSMC that occurred during speech production and perception (S2, cf. Fig. 1B,C). Significant high-gamma RSMC occurred in a number of areas implicated in language^43^, including Broca’s area, the premotor cortex, the inferior parietal cortex, and the temporal cortex.

Overlaps between speech production and perception were spatially focalized: They mostly occurred in small parts of the svPMC and its neighborhood along the central sulcus, in Broca’s area, and in the temporal cortex. Of all anatomical areas, overlaps between speech production and perception were most consistently observed in the premotor cortex and in adjacent parts of the central sulcus. In general, spatial overlaps between both conditions agreed well with mouth motor and speech-essential areas identified using ESM in the frontal, parietal, and temporal lobes (Suppl. Table 1).

#### Anatomical description of the aSPPO region

**Figure 3:**
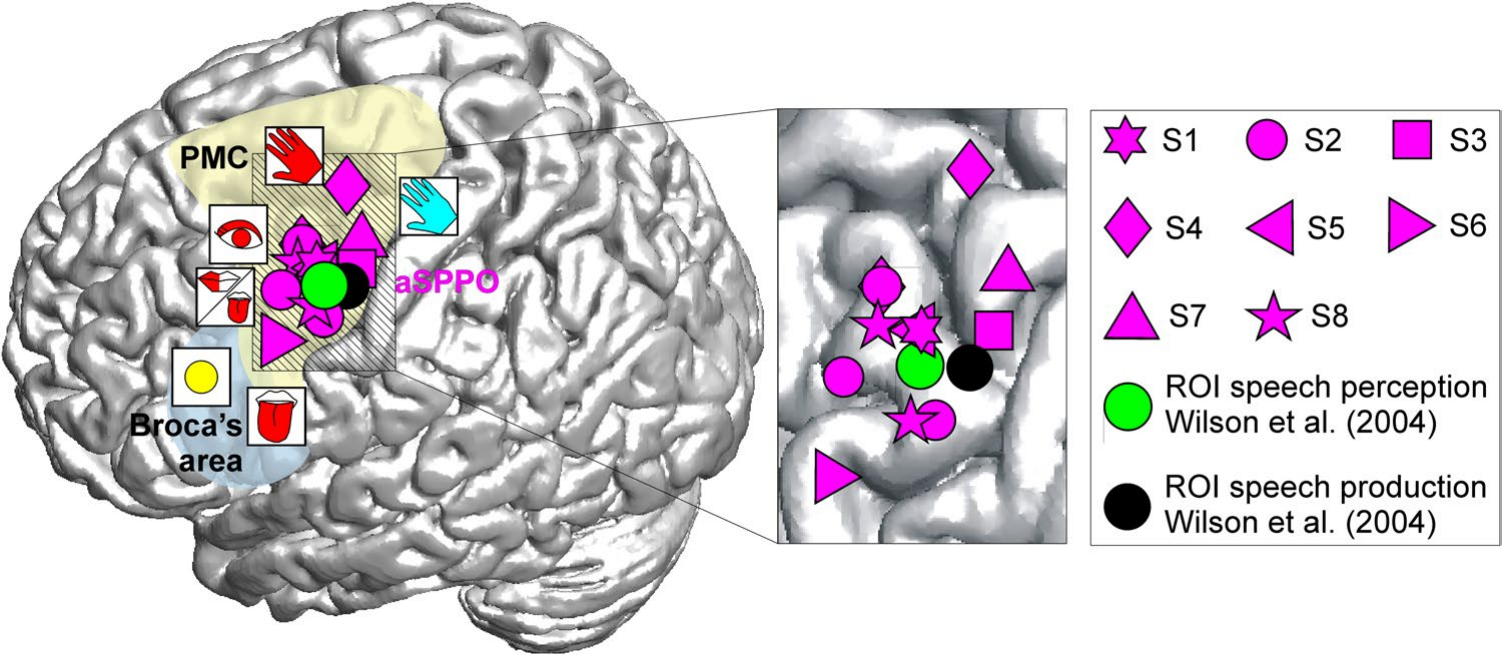
Spatial properties of neuronal activity in the aSPPO region. All electrodes overlying the aSPPO region are visualized on a standard brain based on their MNI coordinates (Suppl. Table 1). Each magenta symbol represents one electrode; different shapes depict different subjects (S1–S8, see legend). The visualized overlap between speech production and perception lies in the superior part of the ventral premotor cortex with mouth motor properties. The yellow and blue overlays schematically indicate the locations of the premotor cortex (PMC) and of Broca’s area, respectively. ESM effects in the neighborhood, visualized using the same conventions as in Fig. 1A, show that the aSPPO region lies juxtaposed between ESM-defined speech-essential, eye motor, hand motor, and hand sensory areas. Speech production and perception areas defined in an fMRI group analysis by Wilson *et al*.^15^ are also shown as black and green circles, respectively. The striped area on the standard brain in which the aSPPO effects took place is magnified on the right. Note that some effects have a strong spatial overlap between subjects due to the similar MNI coordinates in the x and y planes.

The anatomical location of the aSPPO region was in accordance with the position of an overlap in the premotor cortex between speech production and perception that was first reported in an fMRI study by Wilson and colleagues^15^ (3.5 mm 3D distance between (i) mean center of mass of fMRI activations for speech production and perception and (ii) center of mass of our aSPPO electrode positions). Figure 3 shows the locations of aSPPO effects in our study, together with the results reported in^15^.

### Different temporal and frequential properties of activity in the aSPPO region between speech production and perception

Once we had established that a robust overlap exists between expressive and receptive speech in the articulatory cortex during real-world conversations, we were interested in characterizing the neuronal activity in the aSPPO region in more detail. To this end, we estimated the differences in the onset of the earliest significant activation and in the maximum response magnitude between anatomical areas within each condition and within anatomical areas between conditions.

#### Timing of neural activity during speech production and perception

**Figure 4:**
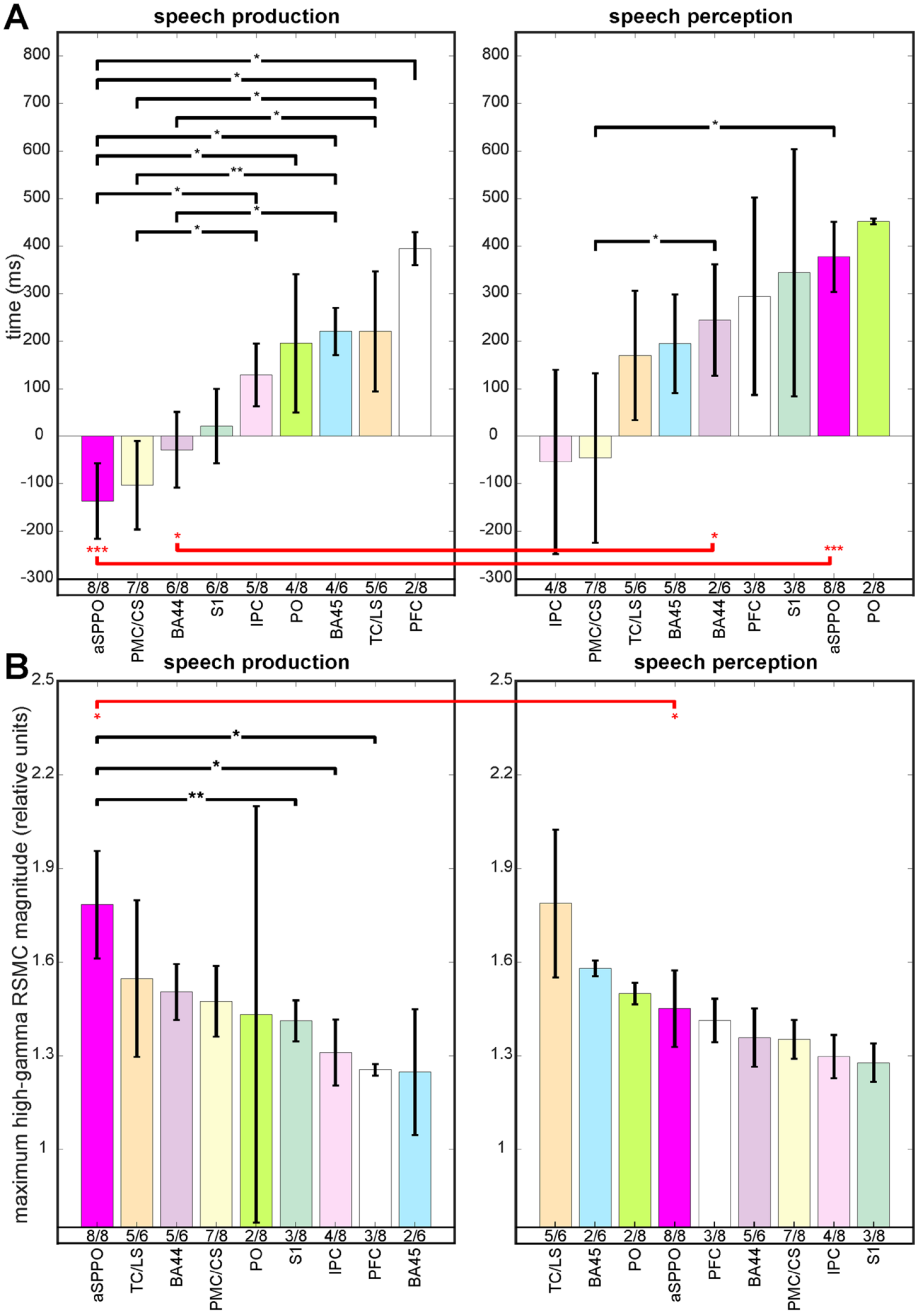
A comparison of the earliest time points of significant activation (**A**,**B**) of the maximum high-gamma response magnitudes between the different anatomical areas within each condition and within each anatomical area between conditions. The height of the colored vertical bars show the earliest timing (**A**) and the maximum high-gamma response magnitude values (**B**) for all frequency bands together, averaged over all electrodes with significant RSMC (Wilcoxon sign test, FDR-corrected at q = 0.005) in each anatomical area of each subject, then averaged in the respective anatomical area (color-coded, abbreviations for the anatomical areas are below the bars) over subjects (see Methods). The left panels of (**A**,**B**) show effects observed in speech production, and the right panels show effects related to speech perception. The number of subjects with significant RSMC in the respective area (those with significant effects in the given area/those with electrode coverage of the respective area) is indicated below the vertical colored bars. The whiskers at each colored bar show the standard errors of the median obtained using a bootstrapping procedure (see Methods). Note that the number of values per anatomical area was often small (2–8 subjects with effects in the given area), so one should not overestimate the importance of the bootstrapping-approximated whisker length with regard to the outcome of statistical testing. (i) Significant differences between anatomical areas within the same condition (Wilcoxon rank sum test, uncorrected) are indicated by black brackets, and (ii) significant differences within the same anatomical area between conditions (Wilcoxon rank sum test, uncorrected) are indicated by red brackets. The height of the p-values is coded by the number of (i) black and (ii) red stars, one star: p < 0.05, two stars: p < 0.01, three stars: p < 0.001.

Our analysis of the earliest time points of significant activation in each cortical area and speech condition revealed temporal differences between areas (Figs 2, 4A, Suppl. Table 2). These were particularly pronounced in speech production, whereas involvement of the different anatomical areas occurred more simultaneously during perception. Activity underlying speech production in the aSPPO region started significantly earlier than in a number of other anatomical areas (in the prefrontal cortex, in the temporal cortex and on the lateral sulcus, in Brodmann area (BA) 45, in the parietal operculum, and in the inferior parietal cortex, see left panel in Fig. 4A). It proceeded with responses in the premotor cortex accompanied by activation of BA44 and of the primary somatosensory cortex at the earlier stages of processing. This activity was followed by approximately simultaneous involvement of several other areas, such as the inferior parietal cortex, the parietal operculum, BA45, the temporal cortex, and ca. 200 ms later by the prefrontal cortex. Interestingly, BA44 was activated significantly earlier than BA45, possibly reflecting functional differences between these compartments of Broca’s area^44^.

In speech perception, earliest activations in BA44 and in aSPPO occurred significantly later than those in the non-aSPPO premotor cortex and on the central sulcus. Activation in all other areas did not show a systematic sequential order (right panel of Fig. 4A, Suppl. Table 2). Significant activations in the inferior parietal cortex and/or in the non-aSPPO premotor cortex and the central sulcus were observed prior to the onset of speech perception. In an attempt to find an explanation to this surprising finding, we looked up, what functional roles the electrodes with this early activation showed in ESM. They lay in the eye motor cortex or in the immediate proximity of the eye motor cortex (not visualized). These effects thus possibly reflect eye movements, which are known to occur prior to speech for mediation of turn transitions in real-world conversations^45^. The effects in the inferior parietal cortex may be related to the processing of communicative intentions^46^.

A within-area statistical comparison between speech production and perception revealed that aSPPO and BA44 had most pronounced temporal differences between conditions. These areas may thus be good candidates to distinguish between speech production and perception in real-world brain-computer-interface applications^47^. Note, however, that our analysis of differences between speech production and perception is based on values that could be obtained from a small population of subjects. Our observation that speech production may be more sequentially organized, whereas speech perception appears to involve a more simultaneous structure of activation of the different cortical areas, will thus need validation in a larger population of subjects.

#### Response magnitudes during speech production and perception

Differences in the maximum magnitude of neuronal activity between anatomical areas were most pronounced during speech production (left panel of Fig. 4B, Suppl. Table 3), where the aSPPO region showed the highest maximum high-gamma RSMC of all areas. A comparison of response magnitudes between areas in speech production revealed that aSPPO was the only region with significant differences from other areas. It was activated more strongly than the prefrontal cortex, the inferior parietal cortex (Wilcoxon rank sum test, both differences were significant with p < 0.05), and the primary somatosensory cortex (Wilcoxon rank sum test, p < 0.01). Maximum response magnitudes in speech perception were generally comparable with those during speech production. Differences between anatomical areas, however, were not as disparate, since no significant difference between areas within this condition could be observed. Maximum response magnitudes in the aSPPO region during speech perception reached 82% of those during speech production. Of all anatomical areas, only aSPPO showed significant differences in the maximum response magnitude between conditions (Wilcoxon rank sum test, p < 0.05, Fig. 4B). This may suggest that fewer neurons were active during perception than during production in this region, and that this difference is topographically specific.

### Role of acoustic background noise

**Figure 5:**
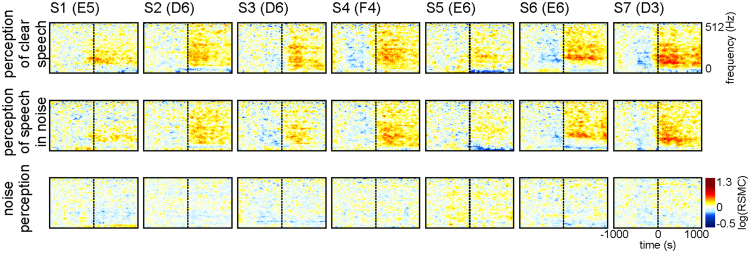
Typical examples of cortical responses in the aSPPO region during clear (top row), noise-embedded (middle row) speech perception, and during perception of loud non-speech acoustic background noises without concurrent speech (bottom row; S1–S7). All conventions as in Fig. 1B,C, electrode names are included in brackets after the subject’s ID (cf. Suppl. Table 1).

To address the hypothesis that perception of noise-embedded speech may require enhanced activity in the aSPPO region, we compared non-experimental clear-speech and speech-in-noise perception (Table 1 in Methods). The former condition was composed of speech without clearly audible background noise, the latter contained time periods in which loud non-speech acoustic background noise roughly coincided with speech perception. The same RSMC analyses as for speech production (Fig. 1B) and perception (Fig. 1C) were conducted (first and second rows of Fig. 5, respectively). As can be seen from Fig. 5, our results do not support higher levels of activation in the aSPPO region during noise-embedded speech: RSMC in both conditions are roughly the same. Visual inspection of the RSMC may even suggest a contrary effect (e.g., electrode E5 in S1 or electrode D3 in S7 with stronger high-gamma responses in clear speech). Differences in the maximum RSMC between clear speech vs. speech in noise in the aSPPO region were not significant (two-tailed Wilcoxon rank sum test, FDR-corrected at q = 0.05) in any of the three investigated high-gamma frequency bands. An additional analysis of non-experimental only-noise perception data (third row in Fig. 5), composed of loud non-speech acoustic background noise (Table 1 in Methods), yielded no high-gamma RSMC effects similar to those during speech perception, and the RSMC responses at aSPPO electrodes in this condition were not significant (two-tailed Wilcoxon sign test, FDR-corrected at q < 0.005).

**Table 1:** Subject details.

S.	age	sex	handedness/ language lateralization	grid location	seizure onset zone	№ hours	№ trials speech production	№ trialsspeech perception	№ trialsnoise perception
S1	40	M	R/B	L fp	L fm	18/26/35	83	137/64/56	106
S2	41	F	L/L	L ftp	L fm	22/48/52	50	146/52/69	107
S3	49	F	R*/B	L ftp	L fm	11/11/14	110	59/53/50	79
S4	39	F	R/L	L ftp	L f pol., L f	10/13/14	46	77/47/54	79
S5	21	M	R/L	L ftp	L fm	13/14/15	85	77/50/54	74
S6	54	M	R/L	L ftp	L f pol.	9/13/15	81	56/61/49	66
S7	27	M	R/L	L fp	L fm	9/7/10	36	61/52/50	72
S8	41	M	R/L	L ftp	L fm	10/12/12	55	106/n.s./n.s.	n.s.

Abbreviations: S.: subject, M: male, F: female, R: right, L: left, R*: right-handed converted from left, f: frontal, p: parietal, t: temporal, m: medial, pol.: polar, n.s.: not studied. № hours, left digit: number of hours in which the speech production events that corresponded to our selection criteria were present, middle digit: same for speech perception, right digit: total number of hours which were chosen for detailed inspection from continuous recordings due to the presence of conversations in them. No trials speech perception, for 3 subsets of data: left value: number of speech perception trials in the perception-production comparison, middle value: number of trials in the acoustic noise-free condition of speech perception, right value: number of trials in the speech perception condition accompanied by strong ambient noises.

## Discussion

This study was inspired by theoretical predictions and ample yet still controversial evidence from experimental research that overlapping speech production- and perception-related neural responses take place in the articulatory motor cortex. We refer to this putative speech production/perception overlap region as aSPPO: ‘a’ for ‘articulatory’, ‘SPPO’ for ‘speech-production-perception-overlap’. Whether a spatial overlap between speech production and perception in the anatomical motor cortex is a representative feature of real-life verbal communication, what properties neuronal activity in this region possesses, and what its role in speech perception is are open questions. It was our aim in the present study to address these questions by testing the following four hypotheses: (i) There is a region in the anatomical motor cortex which is reproducibly activated during natural speech production and perception, (ii) this region has mouth motor properties, (iii) it shows preparatory neuronal activity related to speech production earlier than other cortical areas, and (iv) neuronal activity in this region during perception is modulated by the presence of strong acoustic background noises. The findings of our study lend clear support to the first three hypotheses. Specifically, they demonstrate that the anatomical svPMC corresponding to the dorsal part of the mouth motor cortex is consistently activated during speech perception under real-world conditions of communication. This conclusion draws upon an integrative analysis of ECoG responses across several high-gamma frequency bands combined with precise anatomical information and ESM-based functional mapping of the cortical surface in individual subjects.

Interestingly, no region other than aSPPO was activated in all subjects and both during production and perception. While some areas were still activated with quite good reproducibility (e.g., the premotor cortex outside of aSPPO, BA44, and the primary somatosensory cortex, Fig. 4A), speech-related activation in other areas was less reliable. The prefrontal cortex, e.g., showed significant RSMC only in 2/8 and 3/8 subjects during production and perception, respectively. Based on the available data, a general picture nevertheless emerged: aSPPO was distinguished by a very early activation relative to the onset of speech production. This contrasted with a late recruitment during perception (Fig. 4A). Activation of this region before and after speech onset may reflect functions related to preparation^3^ and online control of speech production, respectively. aSPPO was also the region with the largest activation magnitude during production (Fig. 4B). While aSPPO was most consistently active during both production and perception, it was also the only area with a significant magnitude difference between the two conditions. Thus, of all areas, the aSPPO was, at the same time, showing strongest similarities and strongest differences between speech production and perception. These findings encourage further investigation of these properties of the speech network based on larger samples of individuals.

An additional comparison of clear-speech and speech-in-noise perception did not show a higher level of activation in the aSPPO region in the latter condition (Fig. 5). Our data thus suggest that motor-cortical activity may play a general role in speech perception, and its explanation by a mechanism specialized for adverse listening conditions seems unlikely. Our analysis of the only-noise condition of acoustic perception yielded no significant effects at aSPPO electrodes, indicating that activity in the articulatory cortex during perception might be speech-specific^2,48^.

### Relation to previous experimental findings

Wilson and colleagues^15^ were the first to provide evidence of spatially overlapping activity between speech production and perception in the motor cortex. These researchers investigated brain activity related to overt production of meaningless syllables and listening to the same set of syllables using fMRI. They found that the bilateral BA6, located in the superior-most part of the ventral premotor cortex, was activated in both speech production and perception. The center of mass in the perception-related fMRI data was shifted a few mm anteriorly (see our Fig. 3). Interestingly, the center of mass of the left-hemispheric fMRI overlap found in this experimental study was very close to the aSPPO region delineated in the present study during real-life communication (3,5-mm distance). Wilson and Iacoboni^49^ hypothesized that effects in this overlap area could be interpreted within the theoretical framework of forward modelling (e.g.^50,51^) and concluded that speech perception is a sensorimotor process in which sensory and motor modalities communicate^49,52^. Our study lends support to these earlier findings of shared activity in the svPMC between speech production and perception, showing that such activity takes place not only in neurolinguistic experiments but also in conditions of every-day, real-life communication.

These earlier studies presented novel evidence on the spatial organization of expressive and receptive language. Due to the limitations intrinsic to fMRI as a method for investigating human brain function^53^, however, they were unable to provide a more detailed description of the temporal and functional properties of activity in this region. These studies could not tell if their identified overlaps between speech production and perception in the svPMC lay in the mouth motor cortex, nor could they draw conclusions as to when this region was activated in the time course of speech production^3^. Our findings show that this overlap region lies in the mouth motor cortex as evidenced by ESM (Fig. 3, Suppl. Table 1), and that it shows early activity, reflected in increased ECoG-RSMC across several bands of high-gamma frequencies prior to the onset of speech production (Fig. 4A, Suppl. Table 2).

The aSPPO region in our study was situated between the hand and orofacial subregions of the sensorimotor cortex along the central sulcus (Fig. 3). This region matches well with the overlapping gamma-band activity between speaking and listening to speech under experimental conditions observed in ECoG by Towle *et al*.^33^. While their results were reported without strict statistical correction for multiple comparisons, the overlap in our data persisted in all subjects even with a conservative statistical threshold after FDR correction (two-tailed Wilcoxon sign test, FDR-corrected at q = 0.005). The early activation of the premotor cortex in speech production is also consistent with an ECoG study by Pei *et al*.^54^, which showed that word repetition after visual and auditory presentation is associated with early premotor-cortical responses.

### Mouth motor properties of the overlap region between speech production and perception in the svPMC

In the present study, mouth motor function of the overlap region between speech production and perception in the svPMC could be established with the help of ESM. Stimulation against different reference electrodes may cause different current distributions. We used both monopolar and bipolar stimulation to diminish the likelihood of false positive stimulation results. Our findings firmly establish that the aSPPO in all subjects lay in a mouth motor area (Fig. 3, Suppl. Table 1). ESM also provided useful information for the functional characterization of the neighborhood of the aSPPO region. The functional significance of the surrounding areas was relatively constant: aSPPO bordered dorsally with hand motor and sensory areas, and dorsorostrally with the eye motor cortex in all subjects. Ventrally, the aSPPO region adjoined other mouth motor sites and sometimes also a second hand motor area located in the border region between the ventral premotor cortex and BA44 (Fig. 1, Suppl. Fig. 1). This arrangement, summarized in a schematic functional map in Fig. 3, may provide useful topographical orientation for future studies.

### The choice of signal components may be crucial to the detection of the aSPPO region

Since gamma activity may show task-related differences between subjects^55^, we investigated different gamma frequency bands in order not to miss the overlap between speech production and perception by restricting our analyses to a single frequency band. This was indeed helpful, as differences between subjects could be observed (Suppl. Table 1): While an overlap between speech production and perception in the anatomical motor cortex occurred in all (S2, S4, S7) or at least in two of the three investigated frequency bands (S3, S6, S8), such an overlap in two subjects was found in one frequency band only (100–200 Hz in S1, 200–350 Hz in S5). The variability in the dominant signal component in which overlapping activity between speech production and perception in the motor cortex occurred possibly explains the fact that some previous studies have found none^12^ or non-reproducible patterns of premotor-cortical activation during speech perception^13,14^. Approaches similar to those implemented in the present study may be helpful to account for such inter-individual variability.

### Functional interpretations of aSPPO activity during speech perception

The functional significance of the motor-cortical activity during speech perception is highly controversial. Some researchers interpret it as reflecting evoked articulatory representations^15,56^. Another candidate role is the processing of visemic information^21,57^. Activation of the premotor cortex, however, can also be observed when speech perception takes place in the auditory-only modality^15,21^, and the functional role of premotor activation therefore cannot be restricted to the processing of information from the visual domain.

Other interpretations of the functional significance of such activity during speech perception include generation of forward predictions^49,51^, modulation of auditory speech processing in a top-down manner^58^, sensorimotor integration of speech information within the dorsal pathway^50,59^, recognition of articulatory gestures via the mirror-neuron system^60,61^, or intra- and inter-personal perceptuomotor coupling in a conversation^62^, turn taking being one example of such a phenomenon^63^.

It has also been suggested that activation of motor-cortical regions during speech perception may represent a compensatory mechanism for the retrieval of speech signals under difficult listening conditions, e.g., during speech perception in noise or when the speaker has a foreign accent^4,40^. Experimental reports, however, are divided as to whether activation of the motor cortex only occurs when subjects listen to distorted speech^41^ or also when they attend to clear speech^24^.

To address the possibility that activity in the aSPPO region may be modulated by acoustic background noise during natural communication, we compared the levels of activation during receptive speech in the presence of strong ambient noises with receptive speech without such noises. Enhanced activity in the speech-in-noise condition could be expected if this area were responsible for filtering out the speech-relevant information from the acoustic signal during adverse listening conditions^40^. This was, however, not the case in our data, suggesting that activation in the articulatory premotor cortex during real-world conversations in our data may not be specialized for compensation of acoustic noise in the environment. Note that natural noises in the speech-in-noise condition were shorter than the speech perception periods embedding them (see Methods). Their shorter duration, however, does not explain the complete absence of stronger responses during the speech-in-noise condition. One would expect enhanced noise-related neuronal effects to occur on a shorter time scale. These were completely absent in both speech-in-noise and in noise perception (Fig. 5). We thus consider an explanation of absent enhanced noise-related activity in the motor cortex by the shorter duration of the noises to be unlikely.

Due to the fact that this study was conducted on data from non-experimental, real-life conditions of human behavior, our choice of acoustic phenomena was constrained by the subjects’ situation. They were in a hospital, and most of them had no ward neighbors. It is conceivable that activity in the aSPPO region could be modulated by acoustic phenomena that we could not study due to their seldom occurrence or absence in our materials. Interesting questions, e.g., would be whether the perception of such complex acoustic stimuli as music or of unattended speech during conversations in which the subject is not involved would elicit enhanced neuronal responses in the aSPPO region. These questions may be of relevance for future studies interested in its functional role.

Previous research indicates that activity in the motor cortex can be important for phoneme categorization^27^. It is engaged in phonological discrimination^57^ and correlates with successful categorical perception of consonants in healthy but not in dyslexic subjects^64^. Our study shows that activity in the same articulatory motor area (Figs 1A, 3, Suppl. Fig. 1) starts early in the time course of speech production and ca. 500 ms later in speech perception (aSPPO region in Fig. 4A). In line with^3^, this leads us to tentatively suggest that activity in the aSPPO region might reflect the processing of intentions to produce a certain phoneme in speech production and the retrieval of this intention during speech comprehension. Further evidence to validate this possibility is needed.

### Ecological validity

Ecological validity is an important topic in the context of our research question. A number of single-neuron studies in animals have shown that non-experimental brain activity differs from what can be observed experimentally, and that neuronal processing in complex, natural conditions cannot be reduced to a superposition of responses to simple, artificial stimuli presented in an experimental environment^65^. A body of evidence shows that experimental protocols^12,22^ or loud recording devices^27^ can either evoke^12,22^ or attenuate^27^ cortical activity in the motor cortex during speech perception. Elucidating the neuronal underpinnings of real-world communication is thus required.

Several studies indicate that the choice of the subject group may affect the reproducibility of overlapping activity between speech production and perception, and that a topographical overlap of activity between speech production and perception in the motor cortex is more likely in subjects with especially good working memory^13^ or a high foreign-language proficiency upon presentation of speech in that language^14^. An explanation of our findings by these properties is unlikely: All subjects were rated as ‘normal’ in terms of their cognitive capacities using pre-operative psychological tests, and German was their native tongue. A limitation intrinsic to our approach is that all subjects were neurological patients with epilepsy, and it is conceivable that neuronal activity in these subjects may, to some extent, differ from that in a healthy population. To reduce the possibility of interference of pathological with functional neuronal responses, we only selected subjects in whom the seizure onset zone was outside the ventral premotor cortex we were targeting (Fig. 1A, Suppl. Fig. 1). We also took care to exclude recordings lying within 30 minutes before and after epileptic seizures. A further argument to support the physiological plausibility of our findings is that, whereas the locus of epilepsy differed between subjects, the anatomical and functional properties of the aSPPO region were highly reproducible.

Our study highlights the unique opportunity provided by intracranial EEG recordings to study the human brain during spontaneous, uninstructed, real-world human behavior and shows that such recordings can contribute to resolution of long-standing controversies as addressed here. This out-of-the-lab approach, however, does not seek to replace experimental procedures, which allow for simultaneous control of many variables and can be conducted on healthy subjects. Comparisons between experimental and non-experimental findings are needed, and we anticipate that neurolinguistic research will profit from such comparisons in the years to come.

## Conclusion and Outlook

We show that activity in the articulatory cortex is not an artefact of experimental research but presents a characteristic feature of both real-world speech production and perception. This activity starts very early in the time course of speech production, takes place in speech but not non-speech noise perception, and is not modulated by the presence of naturally-occurring strong acoustic background noises. These findings are consistent with the idea that activity in the articulatory motor cortex might reflect the processing of articulatory intentions^3^, although other functional interpretations are equally possible. Whether activity in this aSPPO region is essential or at least important for comprehension of speech (e.g.^66^) or if its involvement is merely modulatory (e.g.^67^), and what compensatory/other mechanisms possibly take place during perception when the motor system is compromised^68^ remain further questions that need clarification with regard to the enigmatic aSPPO region.

## Methods

### Subjects

Speech production- and perception-related neuronal data were obtained during pre-neurosurgical diagnostics of epilepsy from eight adult subjects (Table 1). All subjects had a normal cognitive status, defined based on pre-operative psycholinguistic tests, and all spoke German as a mother tongue. Eloquent language areas were identified with the help of preoperative fMRI testing and electrocortical stimulation mapping (ESM). They were found in the left hemisphere of all subjects, in two of whom (S1, S3) they were bilaterally organized to frontal and temporal cortices. All subjects gave written informed consent that all audio, video and neuronal data gathered in the time period of pre-neurosurgical diagnostics would be made available for scientific investigation, and the Ethics Committee of the University Medical Center Freiburg had approved the protocol for subject recruitment. All methods were performed in accordance with the relevant guidelines and regulations. The locations and numbers of electrodes were defined solely by the subjects’ individual clinical needs. This study was performed retrospectively upon completion of pre-neurosurgical procedures, and its interests did not affect the course of diagnostics.

### ECoG acquisition and trial selection

Electrocorticography (ECoG) grids of 8 × 8 platinum/stainless-steel electrodes (4-mm diameter, 10-mm centre-to-centre inter-electrode distance) were implanted on the surface of the left fronto-temporo-parietal cortex. ECoG was recorded 24/7 over the entire period of pre-neurosurgical diagnostics of one to two weeks with a clinical AC EEG-System (IT-Med, Germany) at a 1024-Hz sampling rate using a high-pass filter with a cutoff frequency of 0.032 Hz and an antialiasing, low-pass filter at 379 Hz. 25-frame-per-second digital video and audio data were recorded synchronized with ECoG as a part of the diagnostic procedure. Each video frame corresponded to ca. 40 ECoG data points. All ECoG data points were assigned to the concurrent video frame. The neuronal recordings were not downsampled to this resolution, as this would have resulted in losing much of the temporal and hence frequency resolution. ECoG recordings were acquired and visualized together with the audiovisual materials using the Coherence PSG 299 System (Deltamed, Paris). This software allows identifying behavioral and/or neuronal events and their post-hoc tagging in the ECoG signal.

Audiovisual data were manually screened to select recordings during which the subjects were alert, conscious and engaged in real-world face-to-face conversations with visitors or medical staff (Table 1: right-most entries in column 7). Speech production and perception were defined from the point of view of the subject: ‘Production’ refers to the subject’s own overt speech and ‘perception’ refers to the overt speech of other individual discourse participants. Speech perception data were selected only from conversations in which the subjects were direct participants. Recordings during overlapping talk and during conversations in the background were not analyzed. Speech production and perception events were detected by audiovisual inspection of the audio-video data and tagged at their onset in Coherence based on their aural identification by the investigator frame by frame. Since psycholinguistic literature shows that pauses >200 ms are perceived by humans as ‘pauses’ in a conversation^69^, a single epoch of speech production or perception was defined by the absence of such pauses in it. Speech production and perception were annotated at their onsets if the duration of the respective events was at least 1000 ms. The resulting speech production and perception trials were analyzed in the time periods of -1000 to 1000 ms relative to their onset to make sure that speech production and perception in all trials were taking place through the second 1000 ms of this time window. Speech production and perception events were discarded when accompanied by visible arm, leg, trunk, head movements or by loud non-speech background noises (e.g., objects falling/being moved, phone ringing, people coughing etc.) in the time period of at least 1000 ms before and 1000 ms after onset. Recordings lying ≤30 min before and/or after epileptic seizures were excluded to minimize the impact of pathological activity on the ECoG signal. ECoG signals from all channels of the grid were re-referenced to a common average by subtracting the mean over all channels from each channel at each sampled data point.

### Analysis of speech with background noise

One hypothesis to be tested was that the presence of noise during auditory speech perception might enhance activity in the motor cortex due to stronger recruitment of feed-forward prediction mechanisms used to reconstruct speech in noisy environments during natural communication^4,40^. To address this possibility, we collected additional speech perception events which took place in noise-free conditions of speech perception (9^th^ column, entries 2 from the left in Table 1) or in the presence of loud non-speech background noises (9^th^ column, entries 3 from the left in Table 1). Trials in the selected noise-free condition were mostly a subset of the speech perception data described above, and they were identified by discarding trials with low-volume yet audible background noises (e.g., people sighing, quiet steps outside the ward, etc.) in the speech perception data used for the perception-production comparison. These subsets of speech perception data were gathered in S1–S7, and S8 was not evaluated due to the limited amount of suitable material. The speech-in-noise data were collected as follows. Based on a visualization of the auditory signal in ELAN 3.0 (available from http://tla.mpi.nl/tools/tla-tools/elan/elan-old-versions), we first identified noises with high amplitude of the auditory signal. Then, we inspected these for the presence or absence of simultaneous receptive speech. If the other aforementioned inclusion criteria applied (i.e., no movements or speech production of the subjects accompanied these auditory events 1000 ms before and after their onset plus if the duration of the speech perception event was at least 1000 ms), and if noise onsets took place around the onset of speech perception, the speech-in-noise epochs were selected and tagged in the ECoG data using the same procedure as described above. We originally intended to collect speech-in-noise-perception epochs with post-onset noises which were at least 1000 ms long. Since naturally-occurring noises proved only rarely that long, however, the inclusion threshold had to be lowered to at least 200-ms noise duration to be able to collect a sufficient number of trials^4^ from the available data (6^th^ column in Table 1).

### Analysis of non-speech noise

To find out if activity in the motor cortex also took place during perception of non-speech noises, we collected and analyzed ECoG data during the perception of loud background noises (last column in Table 1). The same selection criteria as for the noises in the speech-in-noise condition apply.

### Assignment of electrodes to functional areas

To delineate the locations of the individual functional areas, ESM was performed using an INOMED NS 60 Stimulator (INOMED, Germany) that was temporally aligned with the ECoG recording system. The electrical stimulation artefacts were directly and clearly visible in the ongoing ECoG recordings. Trains of 10-s pulses at 50 Hz with alternating-polarity square waves of 250 μs were applied to non-overlapping pairs of neighboring electrodes. Monopolar stimulation was performed against a functionally-neutral intracranial reference channel to spatially resolve the effect observed upon bipolar stimulation. Stimulation intensity was gradually increased until the induction of a motor effect up to 15 mA or up to 18 mA while testing speech functions. An electrode was attributed a motor, sensory, and/or speech function if the respective effect occurred at any of the tested stimulation intensities. These functional effects were observed within the several first seconds after stimulation or during it, and they were annotated regardless of the exact timing of the effect relative to stimulation onset. An electrode was labelled as functionally neutral if no functional effect was observed when the maximum current intensities were applied. Fig. 1A provides ESM results in S2, and Suppl. Fig. 1 offers an overview of the data from S1, S3–S8. Additional details on the functional assignment of electrodes via ESM are available elsewhere^30^.

Both motor effects of body parts relevant to articulation and transient speech production and/or perception effects can be considered as potentially important for language functions. The former effects were defined whenever ESM elicited a movement or impeded a movement of a body part that is involved in articulation (e.g., tongue, jaw, lips, or diaphragm). The latter effects were defined using a battery of six tasks (counting, execution of body commands, naming objects, reading, repetition of sentences, and a Token Test)^70^ aimed to test whether the subject is capable of understanding and/or producing language upon ESM. As opposed to mouth motor effects, transient post-stimulation impairments in speech production and/or comprehension (e.g., if a person is no longer able to name days of the week in a correct sequence) are interpreted as indications that the underlying cortical area is essential for cognitive language functions^70^.

Delineation of language-essential areas with ESM, however, can be challenging due to the following reasons: On the one hand, it is conceivable that not all ESM-identified mouth motor electrodes are relevant to articulation and that some of them may support other functions (e.g., smiling or eating). On the other hand, some electrodes without language or mouth motor functions in ESM may still record neuronal activity from areas that are important for language^4^. Keeping in mind these considerations, we defined potentially articulation-relevant areas as follows: they had to show both significant high-gamma responses during speech production and mouth motor effects upon ESM.

### Assignment of electrodes to anatomical areas

As in our previous studies^28,30,32,71^, we used a hierarchical probabilistic method which relies on structural post-implantation images to assign electrodes to anatomical areas. T1-weighted magnetic resonance images (MRIs) with full-head coverage were acquired in all subjects using a 1.5-T Vision MRI scanner (Siemens, Erlangen, Germany) with a magnetization-prepared rapid-acquisition gradient-echo sequence at a resolution of 1 × 1 × 1 mm. These data were converted into a Matlab-compatible format with MRIcro^72^ and spatially normalized to the standard Montreal Neurological Institute (MNI) template in SPM5^73^, anatomy toolbox v17. We visualized the MRI data in 3D with the help of custom Matlab-based software, identified and manually marked each electrode as well as the individual positions of the central and lateral sulci. The electrodes were first assigned to the frontal, parietal, and temporal lobes depending on their positions relative to the sulci in the respective subject. A probabilistic atlas system^74^ was used to assign electrodes to most likely anatomical areas of the cortical surface within each lobe based on their MNI coordinates. Additional information on this procedure is available in^30^. Note that our anatomical assignment results based on the MNI coordinates (Suppl. Table 1) may deviate from those obtainable, e.g., using SPM alone due to the fact that we account for individual anatomical landmarks and make orthogonal projections of electrode positions outside of the standard brain onto the SPM-normalized cortical surface. The original MNI coordinates prior to this projection are reported in Suppl. Table 1. Also, note that assignments to the parietal operculum refer to the subregions of this anatomical area that extend to the outer brain surface, e.g., to parts of regions OP 4 (PV) and OP 1(SII) as depicted in Figs 4–7 of^75^. Finally, note that, following the conventions of the SPM anatomy toolbox (see also ref.^76^), the primary motor cortex was defined as equal to BA4. In the vicinity of aSPPO, BA4 is situated within the depth of the central sulcus^18^. Since ECoG is recorded from the cortical surface, electrodes are not in direct contact with hand- and face-related BA4; the electrodes anterior to the central sulcus record from the premotor cortex (BA6), which adjoins the primary motor cortex in this direction. Whenever we refer to the anatomical motor cortex in the present study, we mean the premotor cortex including electrodes extending onto the central sulcus for these reasons.

### Spectral analysis and statistics

We calculated time-resolved spectral magnitude values in each of the selected 2000-ms excerpts of the neuronal data using a time-resolved Fourier transformation with a sliding window of 200 ms, a time step of 20 ms and 5 Slepian tapers^77^. The absolute spectral magnitude at each time-frequency bin at each electrode was baseline-corrected. The baseline period corresponded to the first 200 ms of the 1000-ms epoch of silence preceding the onset of the respective trial. The absolute spectral magnitudes in this period were median-averaged over time and frequency, and relative spectral magnitude changes (RSMC) in each condition were calculated by dividing the time- and frequency-resolved absolute spectral magnitude of each trial by its baseline activity.

The RSMC in each subject were then median-averaged over trials in the respective condition. Gamma activity can behave differently in different frequency components of the ECoG signal^78^, and inter-subject variability of gamma response properties can be observed in the same cortical region during the same task^55^. We therefore investigated three high-gamma frequency bands (70–100 Hz, 100–200 Hz, and 200–350 Hz) to ensure that the obtained results did not critically depend on our definition of the gamma-signal component. The statistical significance of RSMC in each condition was tested against the baseline using a two-tailed Wilcoxon sign test implemented in the signtest function in Matlab (Version R2012b). An example of time-resolved RSMC is provided in Fig. 1B,C, and band-averaged RSMC are illustrated for one frequency band in Fig. 2 (S2).

### Timing of neuronal activity during speech production and perception

We exploited the high temporal resolution of ECoG data to test whether activity in the overlap region between speech production and perception in the anatomical motor cortex would occur early in the time course of speech production as predicted by some theoretical accounts^3^. We were also interested to find out, how the timing of activity in the overlap region between speech production and perception in the anatomical motor cortex would compare to that in the other anatomical areas in the production and perception conditions. We computed the earliest time points at which the high-gamma RSMC were significant (Wilcoxon sign test, FDR-corrected at q = 0.005 for multiple comparisons over the number of tested electrodes and time points within each frequency band) in the analyzed time window of [−1000 1000] ms relative to speech production and perception onset. Within each condition, the earliest time point at which the high-gamma RSMC were significant was first identified for each electrode, subject, and frequency band separately. The results of this computation in each subject and frequency band for each electrode belonging to the given anatomical area (not visualized) were median-averaged across all electrodes belonging to this anatomical area (Suppl. Table 2, not visualized). A median over subjects for each area was subsequently calculated (Suppl. Table 2 and vertical colored bars in Fig. 4A). Since the number of significant values across subjects for some areas was small (Fig. 4A and Suppl. Table 2), a bootstrapping procedure was used (csboot function in Matlab, 100 repetitions) to estimate the standard error of the median (vertical black lines in Fig. 4A). Next, we compared the resulting earliest time points of significant activation using the Wilcoxon rank sum test at p = 0.05 (i) between the nine anatomical areas with significant high-gamma RSMC effects within each condition (black brackets with stars indicating the level of significance next to them in Fig. 4A) and (ii) within each of these individual areas between speech production and perception (red brackets with stars indicating the level of significance next to them in Fig. 4A).

### Response magnitudes during speech production and perception

We also estimated the strongest high-frequency response magnitudes (Suppl. Table 3) and compared them between anatomical areas within the two speech conditions and within the same anatomical area between conditions (Fig. 4B). Exactly the same procedure was applied as for the calculation of the earliest time points of significant activation described above. The highest high-gamma response magnitudes of the significant responses were used in this analysis instead of the earliest significant time points. The statistical differences in the maximum RSMC between the different conditions were also assessed using a two-tailed Wilcoxon rank sum test (ranksum function in Matlab). A FDR procedure was applied for each frequency band at q = 0.05 to account for multiple testing over time bins and grid electrodes.

### Data and code availability statement

The data and the code that support the findings of this study are available from the corresponding authors upon reasonable request.

## Electronic supplementary material

Supplementary Material

## References

[1] Miller, K. *Communication theories*. (USA: Macgraw-Hill, 2005).

[2] Liberman AM, Cooper FS, Shankweiler DP, Studdert-Kennedy M (1967). Perception of the speech code. Psychol Rev.

[3] Liberman AM, Mattingly IG (1985). The motor theory of speech perception revised. Cognition.

[4] Tremblay P, Small SL (2011). On the context-dependent nature of the contribution of the ventral premotor cortex to speech perception. Neuroimage.

[5] Pulvermüller F, Fadiga L (2010). Active perception: sensorimotor circuits as a cortical basis for language. Nat Rev Neurosci.

[6] Liberman, A., Cooper, F. S., Harris, K. S. & MacNeilage, P. F. A motor theory of speech perception. *Proc Sp Comm Sem*, *Stockholm* (1962).

[7] Halle M, Stevens KN (1962). Speech recognition: a model and a program for research. IRE Trans Inform Theory.

[8] Fowler CA (1986). An event approach to the study of speech perception from a directrealist perspective. J Phon.

[9] Fowler, C. A. Speech perception: direct realist theory. In *The Encyclopedia of Language and Linguistics* 4199–4203 (Oxford: Pergamon, 1994).

[10] Pulvermüller, F. *The Neuroscience Of Language: On Brain Circuits Of Words and Serial Order*. (Cambridge: Cambridge University Press, 2003).

[11] Pulvermüller, F. & Fadiga, L. Brain language mechanisms built on action and perception. In *Handbook of Neurobiology of Language* 311–324 (Amsterdam: Elsevier, 2016).

[12] Menenti L, Gierhan SME, Segaert K, Hagoort P (2011). Shared language: overlap and segregation of the neuronal infrastructure for speaking and listening revealed by functional MRI. Psychol Sci.

[13] Szenkovits G, Peelle JE, Norris D, Davis MH (2012). Individual differences in premotor and motor recruitment during speech perception. Neuropsychologia.

[14] Shimada K (2015). Fluency-dependent cortical activation associated with speech production and comprehension in second language learners. Neuroscience.

[15] Wilson SM, Saygin AP, Sereno MI, Iacoboni M (2004). Listening to speech activates motor areas involved in speech production. Nat Neurosci.

[16] Okada K, Hickok G (2006). Left posterior auditory-related cortices participate both in speech perception and speech production: Neural overlap revealed by fMRI. Brain Lang.

[17] Campbell R (2001). Cortical substrates for the perception of face actions: an fMRI study of the specificity of activation for seen speech and for meaningless lower-face acts (gurning). Brain Res Cogn Brain Res.

[18] Fridriksson J (2008). Motor speech perception modulates the cortical language areas. Neuroimage.

[19] Callan DE (2004). Multisensory integration sites identified by perception of spatial wavelet filtered visual speech gesture information. J Cogn Neurosci.

[20] Ojanen V (2005). Processing of audiovisual speech in Broca’s area. Neuroimage.

[21] Skipper JI, van Wassenhove V, Nusbaum HC, Small SL (2007). Hearing lips and seeing voices: how cortical areas supporting speech production mediate audiovisual speech perception. Cereb Cortex.

[22] Hickok G (2012). The cortical organization of speech processing: feedback control and predictive coding the context of a dual-stream model. J Commun Disord.

[23] Menenti L, Pickering MJ, Garrod SC (2012). Toward a neural basis of interactive alignment in conversation. Front Hum Neurosci.

[24] Möttönen R, Dutton R, Watkins KE (2013). Auditory-motor processing of speech sounds. Cereb Cortex.

[25] Osnes B, Hugdahl K, Specht K (2011). Effective connectivity analysis demonstrates involvement of premotor cortex during speech perception. NeuroImage.

[26] Venezia JH, Saberi K, Chubb C, Hickok G (2012). Response Bias Modulates the Speech Motor System during Syllable Discrimination. Front Psychol.

[27] Schomers MR, Pulvermüller F (2016). Is the Sensorimotor Cortex Relevant for Speech Perception and Understanding? An Integrative Review. Front Hum Neurosci.

[28] Derix J, Iljina O, Schulze-Bonhage A, Aertsen A, Ball T (2012). ‘Doctor’ or ‘darling’? Decoding the communication partner from ECoG of the anterior temporal lobe during non-experimental, real-life social interaction. Front Hum Neurosci.

[29] Crone NE, Miglioretti DL, Gordon B, Lesser RP (1998). Functional mapping of human sensorimotor cortex with electrocorticographic spectral analysis. II. Event-related synchronization in the gamma band. Brain.

[30] Ruescher J (2013). Somatotopic mapping of natural upper- and lower-extremity movements and speech production with high gamma electrocorticography. Neuroimage.

[31] Ball T, Kern M, Mutschler I, Aertsen A, Schulze-Bonhage A (2009). Signal quality of simultaneously recorded invasive and non-invasive EEG. Neuroimage.

[32] Derix J (2014). From speech to thought: the neuronal basis of cognitive units in non-experimental, real-life communication investigated using ECoG. Front Hum Neurosci.

[33] Towle VL (2008). ECoG gamma activity during a language task: differentiating expressive and receptive speech areas. Brain.

[34] Manning JR, Jacobs J, Fried I, Kahana MJ (2009). Broadband shifts in local field potential power spectra are correlated with single-neuron spiking in humans. J Neurosci.

[35] Crone NE (2001). Electrocorticographic gamma activity during word production in spoken and sign language. Neurology.

[36] Ray S, Niebur E, Hsiao SS, Sinai A, Crone NE (2008). High-frequency gamma activity (80–150 Hz) is increased in human cortex during selective attention. Clin Neurophysiol.

[37] Sinai A (2005). Electrocorticographic high gamma activity versus electrical cortical stimulation mapping of naming. Brain.

[38] Crone NE, Boatman D, Gordon B, Hao L (2001). Induced electrocorticographic gamma activity during auditory perception. Brazier Award-winning article, 2001. Clin Neurophysiol.

[39] Penfield W, Boldrey E (1937). Somatic motor and sensory representation in the cerebral cortex of Man as studied by electrical stimulation. Brain.

[40] Callan D, Callan A, Gamez M, Sato M, Kawato M (2010). Premotor cortex mediates perceptual performance. Neuroimage.

[41] Du Y, Buchsbaum BR, Grady CL, Alain C (2014). Noise differentially impacts phoneme representations in the auditory and speech motor systems. Proc Natl Acad Sci USA.

[42] Hochberg Y, Benjamini Y (1990). More powerful procedures for multiple significance testing. Stat Med.

[43] Price CJ (2012). A review and synthesis of the first 20 years of PET and fMRI studies of heard speech, spoken language and reading. Neuroimage.

[44] Heim S, Eickhoff SB, Amunts K (2008). Specialisation in Broca’s region for semantic, phonological, and syntactic fluency?. Neuroimage.

[45] Kendon, A. Some functions of gaze-direction in social interaction. *Acta Psychol* 22–63 (1967). 10.1016/0001-6918(67)90005-4 6043092

[46] Carota F (2010). Neural dynamics of the intention to speak. Cereb Cortex.

[47] Iljina O (2017). Neurolinguistic and machine-learning perspectives on direct speech BCIs for restoration of naturalistic communication. Brain Comput Interfaces.

[48] Liberman, A. M., Cooper, F. S., Harris, K. S., MacNeilage, P. F. & Studdert-Kennedy, M. Some observations on the efficiency of speech sounds. In *Models for the perception of speech and visual form* 68–87 (Cambridge, MA: MIT Press, 1967).

[49] Wilson SM, Iacoboni M (2006). Neural responses to non-native phonemes varying in producibility: evidence for the sensorimotor nature of speech perception. Neuroimage.

[50] Poeppel D, Hickok G (2004). Towards a new functional anatomy of language. Cognition.

[51] Haruno M, Wolpert DM, Kawato M (2001). Mosaic model for sensorimotor learning and control. Neural Comput.

[52] Iacoboni M (2008). The role of premotor cortex in speech perception: evidence from fMRI and rTMS. J Physiol Paris.

[53] Logothetis NK (2008). What we can do and what we cannot do with fMRI. Nature.

[54] Pei X, Barbour DL, Leuthardt EC, Schalk G (2011). Decoding vowels and consonants in spoken and imagined words using electrocorticographic signals in humans. J Neural Eng.

[55] Nourski KV, Steinschneider M, Rhone AE (2016). Electrocorticographic Activation within Human Auditory Cortex during Dialog-Based Language and Cognitive Testing. Front Hum Neurosci.

[56] Watkins KE, Strafella AP, Paus T (2003). Seeing and hearing speech excites the motor system involved in speech production. Neuropsychologia.

[57] Dubois, C., Otzenberger, H., Gounot, D., Sock, R. & Metz-Lutz, M.-N. Visemic processing in audiovisual discrimination of natural speech: A simultaneous fMRI-EEG study. *Neuropsychologia* **50**, (2012). 10.1016/j.neuropsychologia.2012.02.016 22387605

[58] Hickok G (2009). The functional neuroanatomy of language. Phys Life Rev.

[59] Hickok G, Poeppel D (2007). The cortical organization of speech processing. Nat Rev Neurosci.

[60] Rizzolatti G, Arbib MA (1998). Language within our grasp. Trends Neurosci.

[61] Rizzolatti G, Craighero L (2004). The mirror-neuron system. Annu Rev Neurosci.

[62] Fowler, C. A. & Xie, X. Involvement of the speech motor system in speech perception. In *Speech motor control in normal and disordered speech: Future developments in theory and methodology* 1–24 (Rockville, MD: ASHA Press, 2016).

[63] Scott SK, Johnsrude IS (2003). The neuroanatomical and functional organization of speech perception. Trends Neurosci.

[64] Dufor O, Serniclaes W, Sprenger-Charolles L, Démonet J-F (2009). Left premotor cortex and allophonic speech perception in dyslexia: a PET study. Neuroimage.

[65] Hasson, U., Malach, R. & Heeger, D. J. Reliability of cortical activity during natural stimulation. *Trends Cogn Sci (Regul Ed)* **14**, 40–48 (2010). 10.1016/j.tics.2009.10.011 PMC2818432 20004608

[66] Meister IG, Wilson SM, Deblieck C, Wu AD, Iacoboni M (2007). The essential role of premotor cortex in speech perception. Curr Biol.

[67] Sato M, Tremblay P, Gracco VL (2009). A mediating role of the premotor cortex in phoneme segmentation. Brain Lang.

[68] Wilson SM (2009). Speech perception when the motor system is compromised. Trends Cogn Sci (Regul. Ed.).

[69] Walker MB, Trimboli C (1982). Smooth transitions in conversational interactions. J Soc Psychol.

[70] Wellmer J (2009). Multitask electrical stimulation for cortical language mapping: Hints for necessity and economic mode of application. Epilepsia.

[71] Pistohl T, Schulze-Bonhage A, Aertsen A, Mehring C, Ball T (2012). Decoding natural grasp types from human ECoG. Neuroimage.

[72] Rorden C, Brett M (2000). Stereotaxic display of brain lesions. Behav Neurol.

[73] Friston KJ (1994). Statistical parametric maps in functional imaging: A general linear approach. Hum Brain Mapp.

[74] Toga AW, Thompson PM, Mori S, Amunts K, Zilles K (2006). Towards multimodal atlases of the human brain. Nat Rev Neurosci.

[75] Eickhoff SB, Grefkes C, Zilles K, Fink GR (2006). The somatotopic organization of cytoarchitectonic areas on the human parietal operculum. Cereb Cortex.

[76] Geyer S (1996). Two different areas within the primary motor cortex of man. Nature.

[77] Percival, D. *Wavelet methods for time series analysis*. (Cambridge: Cambridge University Press, 2000).

[78] Crone NE, Korzeniewska A, Franaszczuk PJ (2011). Cortical γ responses: searching high and low. Int J Psychophysiol.

