# Supplementary material for: Real-life speech production and perception have a shared premotor-cortical substrate
Source: Sci Rep. 2018 Jun 11;8:8898. doi: 10.1038/s41598-018-26801-x (PMC5995900; doi:10.1038/s41598-018-26801-x)
Supplement: Supplementary file 1 — Supplementary Material [file 41598_2018_26801_MOESM1_ESM.docx]

Supplementary Material

**Real-life speech production and perception have a shared premotor-cortical substrate**

Olga Glanz (Iljina)1-6, Johanna Derix4-6, Rajbir Kaur4,7, Andreas Schulze-Bonhage5,8,9, Peter Auer1-3, Ad Aertsen6,9, Tonio Ball4,5,9

^1^ GRK 1624 ‘Frequency effects in language’, University of Freiburg, Germany

^2^ Department of German Linguistics, University of Freiburg, Germany

^3^ Hermann Paul School of Linguistics, University of Freiburg, Germany

^4^ Translational Neurotechnology Lab, Department of Neurosurgery, Medical Center - University of Freiburg, Faculty of Medicine, University of Freiburg, Germany

^5^ BrainLinks-BrainTools, University of Freiburg, Germany

^6^ Neurobiology and Biophysics, Faculty of Biology, University of Freiburg, Freiburg, Germany

^7^ Faculty of Medicine, University of Cologne

^8^ Epilepsy Center, Department of Neurosurgery, Medical Center - University of Freiburg, Faculty of Medicine, University of Freiburg, Germany

^9^ Bernstein Center Freiburg, University of Freiburg, Germany

* Correspondence to: Tonio Ball or to Olga Glanz, Department of Neurosurgery, University Medical Center Freiburg, Faculty of Medicine, University of Freiburg, Germany, Engelbergerstr. 21, D-79106 Freiburg im Breisgau, Germany. E-mails: [tonio.ball@uniklinik-freiburg.de](mailto:tonio.ball@uniklinik-freiburg.de), olga.glanz@uniklinik-freiburg.de

**Supplementary Figure 1:** Electrocortical stimulation mapping (ESM) results (S1, S3-S8). All conventions as in Fig. 1A. This figure has been made using CorelDraw (version X3). See Suppl. Tab. 1 for more information on the overlaps between speech production and perception.

**Supplementary ESM results**

Our thorough stimulation protocol enabled delineation of adjacent functional areas within the facial motor cortex: We could discern a dorsal-to-ventral sequence of spatially distinct motor responses related to eye, lip, and tongue movement areas along the course of the central sulcus. Mouth motor responses mostly took place in the anatomical ventral motor region, whereas cognitive speech-related effects (i.e., those observed when testing receptive and expressive language functions using a dedicated battery of six tasks, see Methods) occurred predominantly in Broca’s area and in temporo-parietal areas. In two subjects (Suppl. Fig. 1), cognitive speech-related effects were additionally observed in the ventro-caudal part of the prefrontal cortex on the borders to posterior Broca’s area (S4) and to the premotor cortex (S5).

Motor responses predominated in anatomically precentral motor areas, and sensory responses were largely located in the primary somatosensory cortex. Both generally occurred at lower stimulation intensities than cognitive speech-related ESM effects, and some electrodes in areas with sensory responses at or below the stimulus intensity of 2 mA showed motor responses at higher intensities. Fig. 1A contains a summary of all effects combined at all stimulation thresholds in S2, and Suppl. Fig. 1 visualizes the corresponding data for the other subjects. Speech-relevant cortical sites with transient cognitive impairments in language production and/or comprehension upon stimulation (yellow dots in Fig. 1A and Suppl. Fig. 1) typically did not show other functions.

In addition to the effects described above, we also annotated sporadic stuttering effects (magenta dots in S1 and S4 here and S2 in Fig. 1 in the main text) that took place upon stimulation of motor electrodes within 5 s after stimulation. They occurred selectively at electrodes in mouth motor areas identified in monopolar and/or bipolar stimulation. However, since the patients did not always speak within this time period, these additional annotations do not provide an exhaustive functional picture, nor do these contacts play a major role in our analyses on overlapping activity between speech perception and production. Systematic instructions to attempt to speak after each stimulation trial might nevertheless be helpful to obtain a more detailed description of potentially language-relevant effects in ESM protocols for testing speech functions.

**Supplementary Table 1:** Overview of all electrodes with significant speech production/perception co-activations in S1-S8. Summary of structural and functional properties of electrodes with relative spectral magnitude change (RSMC) overlaps for the three investigated bands of gamma activity: 70-100 Hz, 100-200 Hz, and 200-350 Hz (respectively bands 1, 2 and 3 in column five). S.: subject, anat. area: anatomical area, γ: gamma, MNI: standard Montreal Neurological Institute co-ordinates of the electrode centre in the respective plane (X, Y, Z), ele.: electrode name (cf. Suppl. Fig. 1). Structural anatomy information in column two is the same as in Fig. 1A. Last column: electrode names are provided for ease of spatial reference (cf. Fig. 1 and Suppl. Fig. 1), electrode names with the symbol ^#^ indicate all aSPPO electrodes (i.e., the ones lying in both anatomical and functional mouth motor areas, cf. Fig. 3).

| **S.** | **anat.  area** | **monopolar ESM response** | **bipolar ESM response** | **γ  band** | **MNI X** | **MNI Y** | **MNI Z** | **ele.** |
| --- | --- | --- | --- | --- | --- | --- | --- | --- |
| S1 | PMC | lip motor | thumb & index finger sensory | 2 | -50 | -5 | 52 | E5^#^ |
| S2 | CS | lip motor, thumb &  index finger sensory | lip motor | 1, 2, 3 | -53 | -8 | 42 | D5^#^ |
|  | PMC | lip motor | lip motor | 1, 2, 3 | -50 | 2 | 43 | D6^#^ |
|  | PMC | lid motor | cheek & lid motor, hand sensory | 1, 2, 3 | -43 | -2 | 51 | E6^#^ |
|  | PO | tongue motor | tongue motor | 1, 2, 3 | -66 | -4 | 13 | A5 |
|  | BA44 | tongue motor | language | 2 | -61 | 16 | 16 | A7 |
|  | BA45 | language | language | 2, 3 | -58 | 26 | 16 | A8 |
| S3 | PMC | tongue & eye lid motor | lip & eye lid motor | 2, 3 | -51 | -13 | 53 | D6^#^ |
|  | LS | not stimulated | not stimulated | 1 | -71 | -16 | 10 | H4 |
|  | TC | not stimulated | not stimulated | 1, 2, 3 | -72 | -25 | 12 | H5 |
| S4 | TC | not stimulated | language | 1, 2, 3 | -70 | -25 | 10 | A3 |
|  | TC | not stimulated | language | 2, 3 | -70 | -14 | 9 | A4 |
|  | TC | not stimulated | not stimulated | 2 | -64 | 9 | 2 | A6 |
|  | PO | lip motor | lip motor | 1 | -68 | -11 | 26 | B4 |
|  | PMC | lip & neck motor | lip & neck motor | 1 | -47 | 0 | 57 | E5^#^ |
|  | PMC | lip motor | lip motor | 1, 2, 3 | -39 | -10 | 66 | F4^#^ |
| S5 | LS | not stimulated | not stimulated | 1, 2, 3 | -65 | -44 | 21 | B2 |
|  | PMC | lip motor | lip & neck motor | 3 | -52 | -5 | 52 | E6^#^ |
| S6 | BA44 | little finger motor | tongue motor | 2 | -44 | 17 | 34 | D5 |
|  | BA44 | ring finger motor | tongue motor | 2 | -45 | 7 | 39 | D6 |
|  | PMC | tongue motor | tongue & lip motor | 2, 3 | -51 | 4 | 32 | E6^#^ |
|  | S1 | thumb & index finger motor | thumb motor | 2 | -51 | -2 | 32 | E7 |
| S7 | BA44 | language | no effect | 1 | -62 | 16 | 16 | A7 |
|  | S1 | tongue motor | tongue, neck & soft palate motor | 1 | -64 | -19 | 41 | B3 |
|  | PMC | tongue & neck motor | lip motor | 1, 2, 3 | -50 | -15 | 58 | D3^#^ |
| S8 | PMC | tongue & chin motor | chin motor | 1, 3 | -51 | -1 | 53 | B3^#^ |
|  | PMC | tongue & chin motor | thumb & index finger sensory | 1 | -58 | -3 | 44 | C3^#^ |
|  | S1 | thumb sensory | face, lip & thigh motor | 1, 2, 3 | -63 | -15 | 38 | D4 |
|  | LS | no effect | lip, chin & neck motor | 1, 2 | -67 | -10 | 12 | F3 |
|  | LS | no effect | lip, chin & neck motor | 1, 2, 3 | -67 | -22 | 16 | F4 |
|  | LS | no effect | language | 1, 2, 3 | -66 | -32 | 16 | F5 |
|  | IPC | no effect | language | 1, 2, 3 | -65 | -49 | 25 | F7 |
|  | TC | no effect | language | 1, 2, 3 | -65 | -41 | 22 | F6 |
|  | TC | not stimulated | no effect | 1 | -60 | 7 | -6 | G1 |
|  | TC | not stimulated | no effect | 1, 2 | -65 | -4 | -3 | G2 |
|  | TC | not stimulated | no effect | 1, 2, 3 | -68 | -15 | 1 | G3 |
|  | TC | not stimulated | no effect | 1, 2, 3 | -70 | -25 | 6 | G4 |
|  | TC | not stimulated | not stimulated | 2 | -65 | -54 | 0 | H7 |

**Supplementary Table 2: Summary of the earliest onsets of significant activations in each anatomical area of S1-S8.** The median earliest onsets of significant activations were calculated for the conditions of speech production (upper half of the table) and perception (lower half of the table), effects for all high-gamma frequency bands are combined (see Methods). Abbreviations: S. in the horizontal row: number of subjects with effects in the respective area (those with significant effects/those with electrode coverage of the respective area), n.c.: areas which were not covered by electrodes in the respective subject, #: covered areas without significant effects, s.err.(median): standard error of the median, other abbreviations as in Fig. 1. See Fig. 4A for a visualization.

| **cond.  area** | **BA44** | **BA45** | **IPC** | **PO** | **PFC** | **S1** | **aSPPO** | **TC/LS** | **PMC/CS** | **SPC** |
| --- | --- | --- | --- | --- | --- | --- | --- | --- | --- | --- |
| **sp. prod.   S.** | **6/8** | **4/6** | **5/8** | **4/8** | **2/8** | **6/8** | **8/8** | **5/6** | **7/8** | **0/4** |
| S1 | 71 | 196 | # | # | # | # | 146 | n.c. | 46 | # |
| S2 | 46 | 245 | # | -103 | # | -54 | -103 | # | -228 | # |
| S3 | # | 96 | 220 | 594 | 345 | 96 | -54 | 494 | -4 | n.c. |
| S4 | -103 | # | 129 | # | # | 162 | -170 | 96 | -103 | n.c. |
| S5 | # | n.c. | # | # | # | # | -352 | 295 | # | # |
| S6 | -178 | 295 | 46 | 196 | # | -54 | -303 | 220 | -253 | n.c. |
| S7 | 46 | # | 196 | 196 | 445 | 245 | 46 | n.c. | 71 | n.c. |
| S8 | -203 | n.c. | -29 | # | # | -153 | -203 | -54 | -203 | # |
| **median** | **-29** | **220** | **129** | **196** | **395** | **21** | **-137** | **220** | **-103** | # |
| **s.err.(median)** | **80** | **50** | **66** | **146** | **35** | **79** | **79** | **127** | **93** | # |
| **sp. perc.   S.** | **5/8** | **2/6** | **4/8** | **2/8** | **3/8** | **3/8** | **8/8** | **5/6** | **7/8** | **0/4** |
| S1 | # | # | 593 | # | # | # | 195 | n.c. | # | # |
| S2 | 519 | 344 | # | 444 | # | # | 494 | 170 | # | # |
| S3 | # | # | -104 | # | # | # | 444 | 195 | # | n.c. |
| S4 | # | # | # | 460 | 294 | # | 361 | 427 | -237 | n.c. |
| S5 | 245 | n.c. | -54 | # | # | # | 593 | 120 | 195 | # |
| S6 | 195 | 45 | # | # | 593 | 344 | 245 | # | # | n.c. |
| S7 | 443 | # | # | # | # | -254 | 394 | n.c. | 145 | n.c. |
| S8 | 170 | n.c. | -154 | # | 20 | 344 | 45 | -254 | -378 | # |
| **median** | **245** | **195** | **-54** | **452** | **294** | **344** | **0.377** | **170** | **-46** | # |
| **s.err.(median)** | **117** | **104** | **190** | **6** | **208** | **260** | **74** | **136** | **178** | # |

**Supplementary Table 3. Summary of the maximum high-gamma RSMC in each anatomical area of S1-S8.** Conventions as in Suppl. Tab. 2. See Fig. 4B for a visualization.

| **cond.  area** | **BA44** | **BA45** | **IPC** | **PO** | **PFC** | **S1** | **aSPPO** | **TC/LS** | **PMC/CS** | **SPC** |
| --- | --- | --- | --- | --- | --- | --- | --- | --- | --- | --- |
| **sp. prod.   S.** | **6/8** | **4/6** | **5/8** | **4/8** | **2/8** | **6/8** | **8/8** | **5/6** | **7/8** | **0/4** |
| S1 | 1.309 | 1.200 | # | # | # | # | 1.908 | n.c. | 1.867 | # |
| S2 | 1.419 | 1.201 | # | 3.559 | # | 1.538 | 1.638 | # | 1.223 | # |
| S3 | # | 1.983 | 1.261 | 1.371 | 1.226 | 1.217 | 2.660 | 1.613 | 1.474 | n.c. |
| S4 | 1.746 | # | 1.656 | # | # | 1.499 | 1.921 | 2.408 | 1.481 | n.c. |
| S5 | # | n.c. | # | # | # | # | 1.638 | 1.459 |  | # |
| S6 | 1.486 | 1.294 | 1.350 | 1.241 | # | 1.289 | 2.269 | 1.307 | 2.318 | n.c. |
| S7 | 1.522 | # | 1.210 | 1.494 | 1.284 | 1.374 | 1.659 | n.c. | 1.384 | n.c. |
| S8 | -0.203 | n.c. | 1.310 | # | # | 1.449 | 1.439 | 1.547 | 1.471 | # |
| **median** | **1.504** | **1.247** | **1.310** | **1.432** | **1.255** | **1.411** | **1.783** | **1.547** | **1.474** | # |
| **s.err.(median)** | **0.086** | **0.18** | **0.064** | **0.508** | **0.022** | **0.070** | **0.172** | **0.237** | **0.142** | # |
| **sp. perc.   S.** | **5/8** | **2/6** | **4/8** | **2/8** | **3/8** | **3/8** | **8/8** | **5/6** | **7/8** | **0/4** |
| S1 | # | # | 1.435 | # | # | # | 1.597 | n.c. | # | # |
| S2 | 1.232 | 1.614 | # | 1.545 | # | # | 1.308 | 1.249 | # | # |
| S3 | # | # | 1.166 | # | # | # | 1.402 | 1.934 | # | n.c. |
| S4 | # | # | # | 1.453 | 1.574 | # | 1.686 | 2.238 | 1.309 | n.c. |
| S5 | 1.332 | n.c. | 1.349 | # | # | # | 1.161 | 1.787 | 1.286 | # |
| S6 | 1.628 | 1.545 | # | # | 1.413 | 1.347 | 1.499 | # | # | n.c. |
| S7 | 1.523 | # | # | # | # | 1.278 | 1.690 | n.c. | 1.395 | n.c. |
| S8 | 1.358 | n.c. | 1.245 | # | 1.400 | 1.176 | 1.172 | 1.488 | 1.538 | # |
| **median** | **1.358** | **1.580** | **1.297** | **1.499** | **1.413** | **1.278** | **1.450** | **1.787** | **1.352** | # |
| **s.err.(median)** | **0.110** | **0.026** | **0.070** | **0.031** | **0.074** | **0.065** | **0.118** | **0.232** | **0.060** | # |
